# Analysis of Toll-Like Receptors in Human Milk: Detection of Membrane-Bound and Soluble Forms

**DOI:** 10.1155/2019/4078671

**Published:** 2019-12-04

**Authors:** Chiara Cattaneo, Alice Caramaschi, Elena Uga, Michela Braghin, Gianluca Cosi, Chiara Peila, Maria C. Strozzi, Miriam Sabatini, Diego Gazzolo, Marcello Manfredi, Maria Cavaletto

**Affiliations:** ^1^Dipartimento di Scienze e Innovazione Tecnologica, University of Piemonte Orientale, 13100 Vercelli, Italy; ^2^S.C. Pediatria, P.O. Sant'Andrea di Vercelli, 13100 Vercelli, Italy; ^3^Terapia Intensiva Neonatale, Azienda Ospedaliera Nazionale SS. Antonio e Biagio e Cesare Arrigo, 15121 Alessandria, Italy; ^4^ISALIT s.r.l., 28100 Novara, Italy

## Abstract

The bioactive and anti-inflammatory role of human milk components has been recognized; active milk components include soluble forms of Toll-like receptors (TLRs). Preterm babies are more susceptible to infections and may succumb to necrotizing enterocolitis (NEC), a gastrointestinal disease which is exacerbated by an excessive inflammatory response after TLR activation. Here, we investigated the presence of Toll-like receptors TLR1/2/4/6 in colostrum and mature milk of women who delivered before (preterm) or after (term) 37 weeks of gestational age, integrating classical immune-related techniques with proteomic LC-MS/MS analysis. We have detected immunoreactivity for TLRs mostly in preterm samples, even for TLR1 and TLR6, until now not described in human milk. We demonstrated the presence of only TLR2 in the milk fat globule membrane, while the immunoreactivity of TLR1/4/6 was ascribed to crossreaction with some interesting milk proteins sharing leucine-rich repeat domains. These results will provide new insights into the definition of the role of TLRs in intestinal immune regulation of the newborns.

## 1. Introduction

Milk is the first food of mammals, providing them nutrients but also protection via immunoglobulins and other immune-related molecules. Milk composition is extremely dynamic, changing its content in nutrient and bioactive factors through the lactation stages (colostrum-transition-mature milk) in order to fulfil the growth needs of the newborn [1]. Milk proteins are classified as caseins, whey proteins, and milk fat globule membrane (MFGM) proteins derived from the apical membrane of the milk-producing epithelial cells. The most abundant proteins are contained in whey and casein fractions, while MFGM proteins represent a minor part (2-4%) of the milk total protein content [2]. However, minor proteins include nonnutrient bioactive factors involved in organism development and immune system maturation. The benefits of human breast milk for human infants, in diminishing mortality and protecting against specific infections during the period of breastfeeding, are well documented ([1] and references therein). Anyway, the contribution of human milk molecules to the development of the newborn's innate and adaptive immune function is still a matter of study. Toll-like receptors (TLRs) are transmembrane glycoproteins, involved in the innate immune response, which recognize conserved molecular structures. TLRs are composed of an extracellular domain with leucine-rich repeats (LRRs), a single-path transmembrane domain, and an intracellular domain called TIR (Toll/IL-1 resistance). The ectodomain is involved in the recognition of ligands, which induce the dimerization of the intracellular domain. TLR2 forms heterodimers with TLR1 and TLR6 and recognizes the broadest range of pathogen-associated molecular patterns (PAMPs) among TLRs, including diacylated and triacylated bacterial lipopeptides and glycolipids such as lipoteichoic acid from Gram-positive bacteria and lipoarabinomannan from mycobacteria. TLR4 requires the association with Myeloid Differentiation Factor 2 (MD-2), a soluble protein that associates with the extracellular domain of TLR4, to recognize the lipopolysaccharides (LPS). TLR activation starts a signaling cascade which leads to nuclear translocation of NF-*κ*B and synthesis of proinflammatory cytokines. Physiologically, TLR signaling is modulated by negative regulators, possibly including soluble forms of the receptor itself. This mechanism avoids the perpetuation of an inflammatory response which could bring irreversible damage of the organism ([3] and references therein).

Babies born before 37 weeks of gestation are defined as “preterm” and present a multifactorial syndrome due to their limited organ development at birth. Preterm babies are more susceptible to infections and may succumb to necrotizing enterocolitis (NEC), a gastrointestinal disease which is exacerbated by an excessive inflammatory response after TLR activation. In this study, we have investigated the presence of TLR1/2/4/6 in breast milk, using both immunodetection techniques and proteomic LC-MS/MS analysis. It has been reported that immunochemical methods applied to a complex substrate such as milk are of concern due to crossreactivity or nonspecific antibody recognition [4]. Until now, soluble forms of TLR2 were immunodetected in breast milk such as 6 isoforms from 20 to 85 kDa [5]; sTLR2 can modulate TLR2 signaling and suppress inflammation [6]. We investigated if it is possible to detect membrane-bound and soluble forms of TLRs by immunoblotting and mass spectrometry. Deeper knowledge of anti-inflammatory molecules, like soluble forms of TLRs (or those present in MFGM), in human milk and their mode of action may help in the development of strategies to prevent infections in premature newborns.

## 2. Materials and Methods

### 2.1. Human Milk Samples

In this study, we investigated the presence of immune-related proteins such as TLRs in colostrum (0) and mature milk (2) of women who delivered before (preterm) or after (term) 37 weeks of gestation. Human milk samples used in this study were provided by Azienda Ospedaliera Nazionale SS. Antonio e Biagio e Cesare Arrigo, Alessandria, and the Milk Bank of Ospedale Sant'Andrea, Vercelli, Italy. The samples (5-10 mL) were not pooled, in order to keep a trace of biological variability, added with a protease inhibitor cocktail (cOmplete, Mini; Roche), aliquoted in 1.5 mL tubes, and stored at -80°C until use.

### 2.2. Milk Fractionation

Milk aliquots were centrifuged at 2000 × *g* for 30 min at 10°C (Fresco 21, Heraeus), and the subsequent two fractions (milk fat globule membranes (MFGMs) and skimmed milk) were analyzed separately. The floating MFGM fraction was prepared as described in [7], transferred to a new tube, and washed three times with NaCl 0.9%, followed by a centrifugation step (3000 × *g*, 30 min, 10°C). MFGM proteins were solubilised from membranes after 1-hour incubation at room temperature in urea 7 M, thiourea 2 M, CHAPS 4%, and DTT 100 mM, then centrifuged at 10000 × *g* for 15 min at 10°C, and the soluble fraction was stored at −20°C until use. The liquid fraction with high protein content, termed skimmed milk, was carefully recovered in order to avoid contamination from other fractions and stored at -20°C until use.

Protein concentration of each fraction was quantified by the method of Bradford [8] at 595 nm with a Spark 10M microplate reader (Tecan). BSA was used as the protein standard. The method was optimized for absorbance readings on a 96-well microplate, mixing 10 *μ*L of buffer/sample/standard dilution with 200 *μ*L of Bradford reagent (Serva).

### 2.3. Immunoreactivity Detection

The fractions of MFGM and skimmed milk were analyzed by SDS-PAGE and immunoblotting against TLR1, TLR2, TLR4, and TLR6. The immunoreactivity to *β*-actin was tested as the internal reference in the same samples. TLR-reactive profiles of preterm and term samples were compared by semiquantitative analysis. Skimmed milk and MFGM fractions were also incubated with anti-TLR antibodies absorbed in a multiwell plate, using a modified ELISA. After removal of the liquid, proteins interacting with the antibody were eluted and recovered. Immunoreactive bands excised from membranes and/or the corresponding SDS-PAGE bands and eluted fractions from the ELISA were digested with trypsin and analyzed by LC-MS/MS using a DDA approach.

#### 2.3.1. SDS-PAGE

Proteins from skimmed milk and MFGMs were analyzed by SDS-PAGE. The samples (20 *μ*g) were mixed with an equal volume of Laemmli reducing sample buffer and boiled. Skimmed milk proteins were separated on a 12.5% polyacrylamide gel, while MFGM proteins were separated on a 10% gel. A molecular weight protein standard was also loaded (Precision Plus Protein™ Dual Color Standards, Bio-Rad, CA, USA). The gels were run in a Mini Protein Tetra System (Bio-Rad, CA, USA) at 100-120 V. The gels were run in duplicate: one was subjected to Coomassie staining and the other was used for Western blotting. In the first case, the gels were fixed in 40% methanol and 10% acetic acid and stained overnight with Blue Silver [9]. After destaining, the gel images were acquired with a GS-900 densitometer (Bio-Rad, CA, USA).

#### 2.3.2. Western Blot Analysis of TLR1, TLR2, TLR4, and TLR6

After SDS-electrophoresis, proteins were transferred onto a polyvinylidene fluoride (PVDF) membrane in a Mini Trans-Blot cell (Bio-Rad, CA, USA) at a constant voltage of 100 V on ice for about 90 min. The membranes were incubated for one hour at room temperature with a blocking solution of PBS/BSA 5%+NaN_3_, then washed for 5 min in Tris-buffered saline (TBS: 150 mM NaCl, 10 mM Tris-HCl (pH 7.4)), to remove the exceeding BSA. Multiple replicates were run in order to probe different antibodies on the same sample, as reported in Table 1: anti-TLR1 (STJ25862, St John's Laboratory), anti-TLR2, anti-TLR4 (sc-10739, sc-10741, Santa Cruz Biotechnology, Inc.), and anti-TLR6 (PRS3653, Sigma-Aldrich Inc., St. Louis, MO, USA). Each membrane was split into two identical parts, containing the same samples, and one half was probed with an anti-*β*-actin antibody (20536-1-AP, Proteintech). The membranes were incubated overnight at 4°C with a primary antibody, then washed once with Tris-buffered saline-Tween (TBST: 150 mM NaCl, 10 mM Tris-HCl, and 0.1% Tween 20) for 15 min, followed by three washes with TBS (10 min). The membranes were then incubated with an alkaline phosphatase- (AP-) conjugated secondary antibody (1 : 3000 in TBS) (Sigma-Aldrich Inc., St. Louis, MO, USA) at room temperature for 1 hour. The membranes were then washed once in TBST for 15 min and 4 times in TBST for 5 min and left in AP buffer (100 mM Tris-HCl (pH 9.5), 100 mM NaCl, and 5 mM MgCl_2_) for 5 min. The signals were detected with BCIP/NBT (5-bromo-4-chloro-3′-indolylphosphate p-toluidine salt/nitro blue tetrazolium chloride, Sigma-Aldrich Inc., St. Louis, MO, USA), the membrane images were acquired with a GS-900 densitometer (Bio-Rad, CA, USA), and band intensities were measured with Image Lab Software (5.2.1 version, Bio-Rad, CA, USA) and normalized to the expression of *β*-actin for semiquantitative analysis.

#### 2.3.3. Modified ELISA of Skimmed Milk

In order to identify soluble components of skimmed milk bound to anti-TLR1/TLR2/TLR4/TLR6 Abs, colostral skimmed fractions from healthy preterm donors were analyzed with a modified ELISA as described in [10]. An ELISA flat-bottomed 96-well microplate (Pure Grade™, BRAND, Wertheim, Germany) was coated with 200 *μ*L/well of the antibody solution in coating buffer (0.05 M sodium carbonate-bicarbonate (pH 9.8)). The plates were incubated for 4 h at 37°C and washed with TBST. Residual binding sites were blocked with 200 *μ*L/well of 1% BSA and washed in TBST. Samples in TBST were added and left overnight at 4°C. After a wash with TBST, 100 *μ*L of elution buffer (0.1 M Glycin-HCl (pH 2.7)) were added and left for 30 min. Finally, the eluate was collected and neutralized with ammonium bicarbonate 0.1 M and immediately submitted to trypsin digestion.

### 2.4. Trypsin Digestion of Proteins

Before mass spectrometry analysis, protein bands separated on SDS-PAGE, immunoreactive bands detected on PVDF membranes, and eluted fractions from the modified ELISA were submitted to trypsin digestion. Proteins from SDS-gels were in-gel digested as described in [11] with a few modifications. Briefly, the protein bands corresponding to the apparent MW of anti-TLR1/2/4/6 immunoreactive bands were excised from Coomassie-stained gels, destained overnight with 50% methanol and 5% acetic acid. The gel pieces were subjected to a series of shrinking steps in acetonitrile, followed by rehydration with 100 mM ammonium bicarbonate (NH_4_HCO_3_). Proteins were then reduced with 10 mM dithiothreitol (DTT) in 100 mM NH_4_HCO_3_ for 30 minutes at room temperature and alkylated in the dark with 100 mM iodoacetamide in 100 mM NH_4_HCO_3_ at room temperature for 30 minutes. The solution was removed and bands were rinsed in 100 mM NH_4_HCO_3_ for 5 minutes. After the final rehydrating step, gel bands were dried in Concentrator Plus (Eppendorf, Germany). Trypsin (Sequencing Grade, Roche, Germany) was reconstituted and added to gel pieces. The digestion was performed overnight at 37°C. The supernatant was collected in a new vial, and peptides were extracted twice in 50% ACN/0.1% formic acid (FA) for 10 min with an ultrasonic bath. The supernatants were pooled, dried, and stored at −20°C until mass spectrometry (MS) analysis.

Immunoreactive protein bands on PVDF membranes were excised, thoroughly washed with HPLC water, then washed once with 50 mM NH_4_HCO_3_, and submitted to reducing-alkylating steps as described for in-gel digestion. After two washes in 50 mM NH_4_HCO_3_, protein bands were covered with trypsin solution and incubated overnight at 37°C. Trypsin-digested peptides were collected as described above. In the case of fainter bands, 4-5 replicates of the same band were digested and pooled in order to increase the amount of protein to be analyzed.

Eluates from the modified ELISA were reduced, alkylated, covered with trypsin solution as described above, and incubated overnight at 37°C. The digested samples were finally dried in Concentrator Plus (Eppendorf, Germany) and stored at -20°C until mass spectrometry analyses.

### 2.5. Mass Spectrometry Characterization

The peptide digests were desalted on the Discovery® DSC-18 solid phase extraction (SPE) 96-well plate (25 mg/well) (Sigma-Aldrich Inc., St. Louis, MO, USA) prior to the mass spectrometry analysis. The LC-MS/MS analyses were performed with a micro-LC Eksigent Technologies (Dublin, USA) system that included a micro LC200 Eksigent pump with flow module 5-50 *μ*L and a programmable autosampler CTC PAL with a Peltier unit (1.0-45.0°C). The stationary phase was a Halo Fused C18 column (0.5 × 100 mm, 2.7 *μ*m; Eksigent Technologies, Dublin, USA). The mobile phase was a mixture of 0.1% (*v*/*v*) formic acid in water (A) and 0.1% (*v*/*v*) formic acid in acetonitrile (B), eluting at a flow rate of 15.0 *μ*L min^−1^ and at an increasing concentration of solvent B from 2% to 40% in 30 minutes. The injection volume was 4.0 *μ*L. The oven temperature was set at 40°C.

The LC system was interfaced with a 5600^+^ TripleTOF™ system (AB Sciex, Concord, Canada) equipped with DuoSpray™ Ion Source and CDS (Calibrant Delivery System). The mass spectrometer worked in a data-dependent acquisition mode (DDA). Peptide profiling was performed using a mass range of 100–1300 Da (TOF scan with an accumulation time of 100.0 ms), followed by a MS/MS product ion scan from 200 to 1250 Da (accumulation time of 5.0 ms) with the abundance threshold set at 30 cps (35 candidate ions can be monitored per cycle). The ion source parameters in an electrospray positive mode were set as follows: curtain gas (N_2_) at 25 psig, nebulizer gases GAS1 at 25 psig and GAS2 at 20 psig, ionspray floating voltage (ISFV) at 5000 V, source temperature at 450°C, and declustering potential at 25 V.

### 2.6. Protein Database Search

The DDA files were searched using Mascot v. 2.4 (Matrix Science Inc., Boston, USA). Trypsin as a digestion enzyme was specified with 2 missed cleavages. The instrument was set to ESI-QUAD-TOF, and the following modifications were specified for the search: carbamidomethyl cysteines as fixed modification and oxidized methionine as variable modification. A search tolerance of 50 ppm was specified for the peptide mass tolerance, and 0.1 Da for the MS/MS tolerance. The charges of the peptides to search for were set to 2+, 3+, and 4+, and the search was set on monoisotopic mass. The UniProt Swiss-Prot *human* unreviewed database (version 2017.06.21, containing 43234 sequence entries) was used. Only proteins with at least two peptides with individual ion scores > 20 were considered for identification purposes.

### 2.7. TLR1/2/4/6 Sequence Alignment

**Figure 1:**
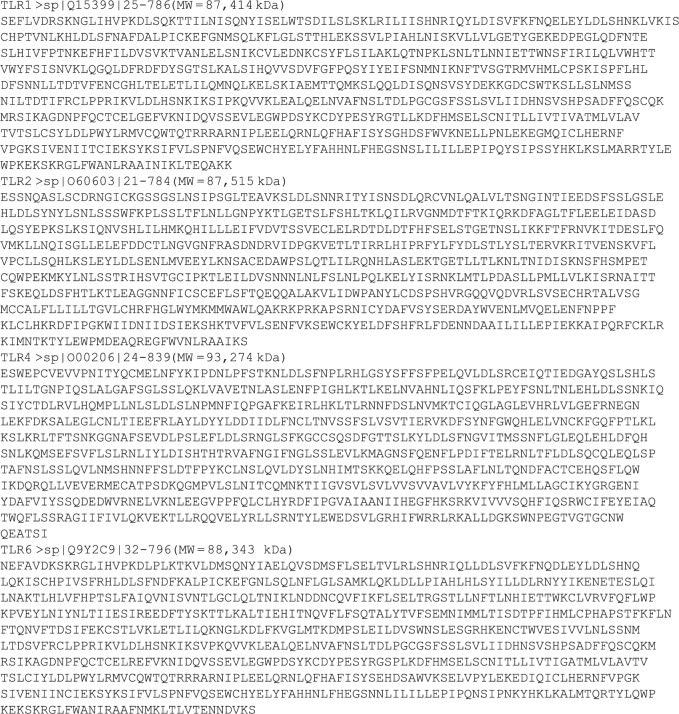
Sequences of TLR1, TLR2, TLR4, and TLR6 mature forms, preceded by UniProt identifier, initial and final amino acid number, and estimated molecular weight.

Sequences of TLR1/2/4/6 mature forms were aligned with Clustal Omega [12] in order to evaluate their homology degree. FASTA sequences of TLR1, TLR2, TLR4, and TLR6 used for the alignment are reported in Figure 1. The alignment of mature TLR1 with TLR6 revealed an identity percentage of 69.4%. TLR4 has the lowest identity compared with the other TLRs (23-25%). TLR2 shows a higher percentage of identity with TLR1 and TLR6 (30%) than with TLR4 (22.7%).

## 3. Results and Discussion

### 3.1. SDS-PAGE Protein Profile

**Figure 2:**
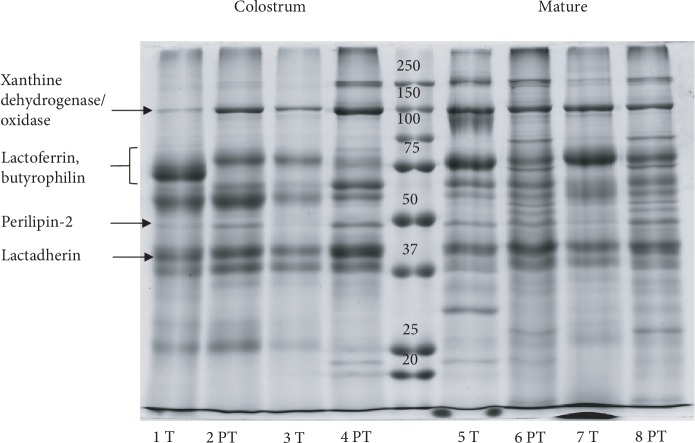
Electrophoretic profile of MFGM proteins in colostrum and mature milk. Gestational age is indicated as “T” (term) or “PT” (preterm). Each number refers to a different milk sample.

**Figure 3:**
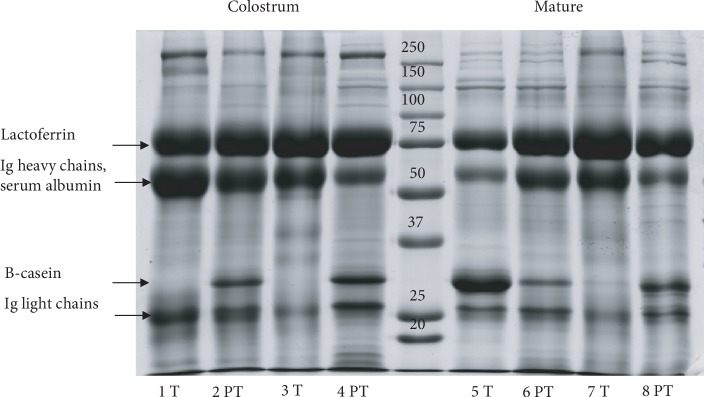
Electrophoretic profile of skimmed milk proteins in colostrum and mature milk. Gestational age is indicated as “T” (term) or “PT” (preterm). Each number refers to a different milk sample.

The protein profile of MFGM and skimmed milk (SM) fractions after SDS-PAGE is shown in Figures 2 and 3, respectively. Milk samples obtained from healthy donors who delivered at term (T) or preterm (PT) and two milk stages (colostrum and mature milk) were analyzed.

MFGM and skimmed milk fractions show a different and characteristic protein profile. Though protein profiles of samples from the same milk fraction are quite similar, it is possible to observe little individual variability, with protein bands of different intensities. The most notable difference is observed between samples of different gestational ages; the colostrum of donors who delivered at term shows the lack of a band related to casein, with regard to preterm samples. The absence of casein in the colostrum of early production is reported in literature [13, 14]. In our case, the difference between term and preterm colostrum could be related to a delayed collection of milk (between the 3rd and 5th day of lactation) in mothers who delivered at preterm.

### 3.2. Analysis of TLR Expression in MFGMs

**Figure 4:**
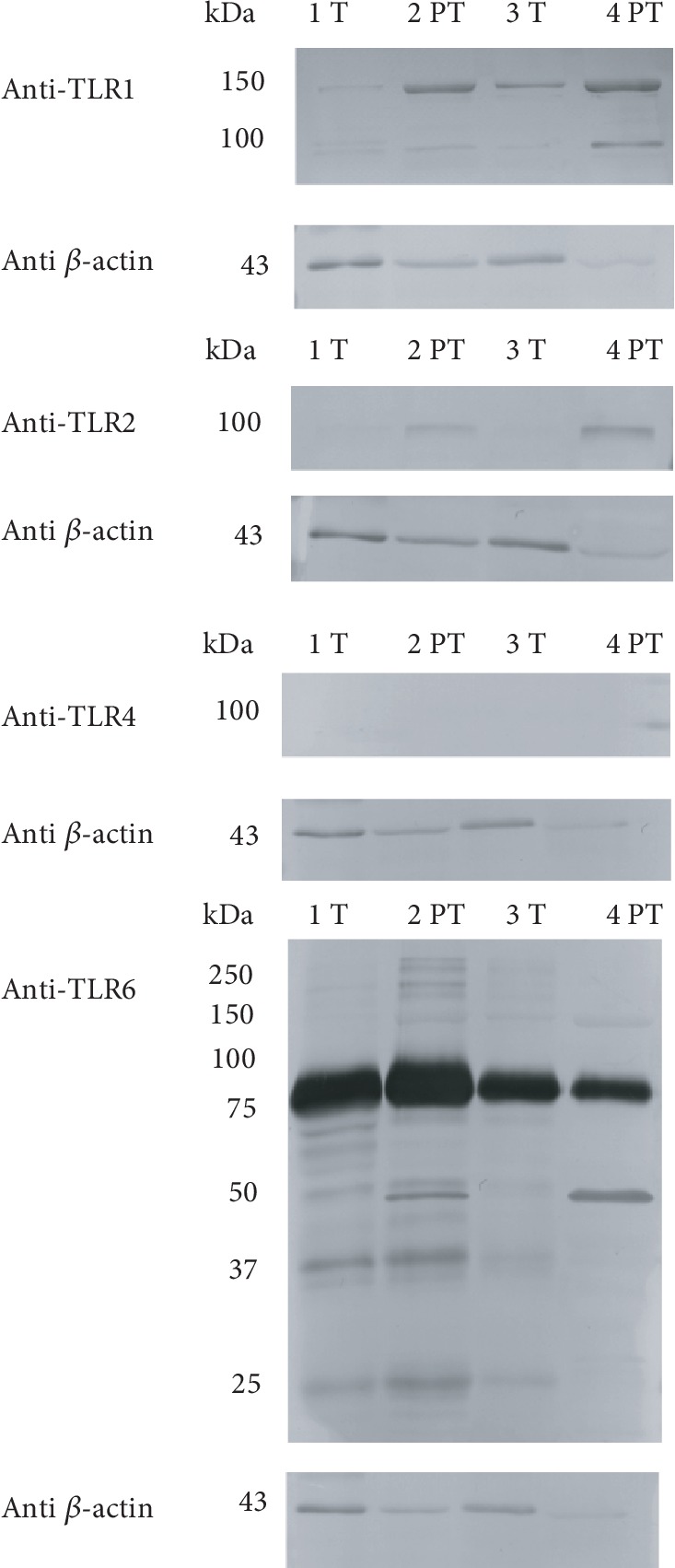
Immunoblot of TLR1/2/4/6 in MFGMs of colostrum. Gestational age is indicated as “T” (term) or “PT” (preterm). Each number refers to a different milk sample. Standard molecular weights are indicated on the left. Each box shows the blot reactivity to a specific anti-TLR antibody and, on the lower side, the same blot incubated with an anti-*β*-actin antibody.

**Figure 5:**
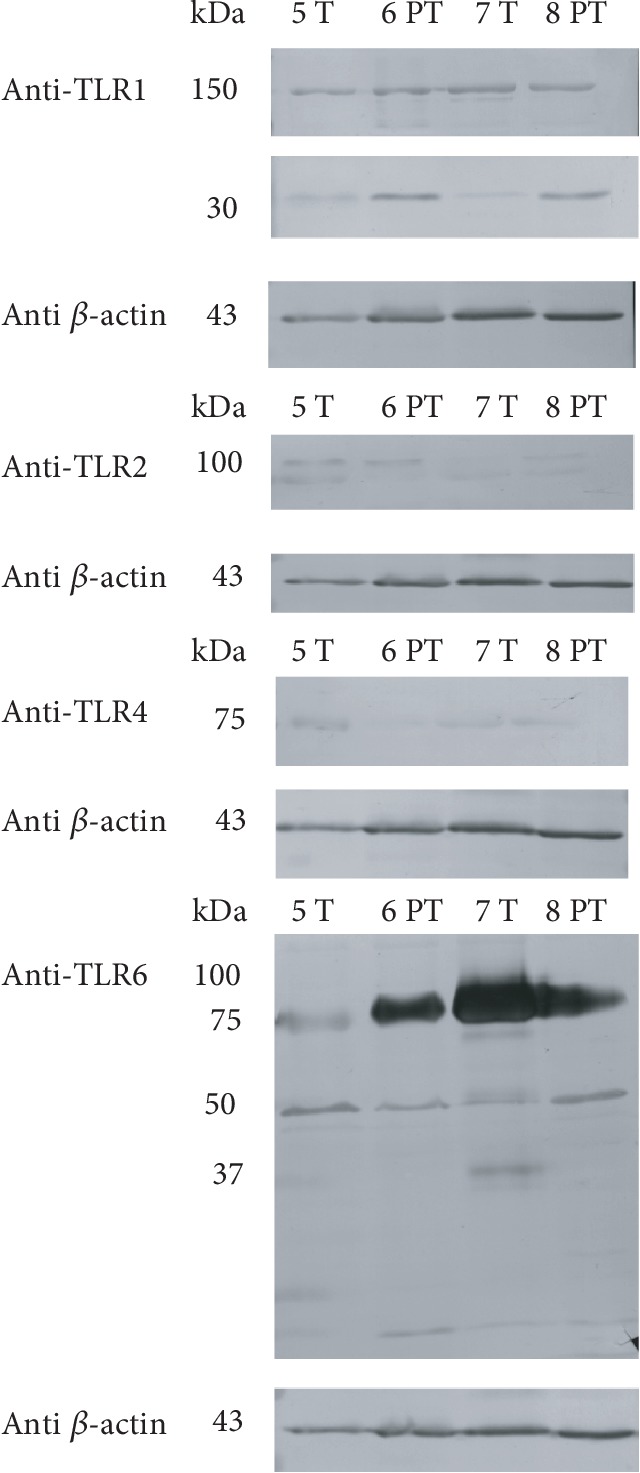
Immunoblot of TLR1/2/4/6 in MFGMs of mature milk. Gestational age is indicated as “T” (term) or “PT” (preterm). Each number refers to a different milk sample. Standard molecular weights are indicated on the left. Each box shows the blot reactivity to a specific anti-TLR antibody and, on the lower side, the same blot incubated with an anti-*β*-actin antibody.

Immunoblots of MFGMs obtained from colostrum and mature milk samples using anti-TLR1/TLR2/TLR4/TLR6 are shown in Figures 4 and 5, respectively. Colostrum showed immunoreactivity versus anti-TLR1 at 150 kDa and 100 kDa, especially in preterm samples. Similarly, anti-TLR2 reactivity is more evident in preterm colostrum, with the appearance of a single band at 100 kDa. We could not observe any reactive band after anti-TLR4 Ab incubation (see also Supplementary [Supplementary-material supplementary-material-1] and [Supplementary-material supplementary-material-1]). Finally, the incubation with anti-TLR6 Ab determined the appearance of a prevalent band at 80-85 kDa and a series of bands with lower molecular weight. Among these, a 50 kDa band is especially evident in preterm milk. The presence of bands around 80-100 kDa is in accordance with the molecular weight of the analyzed TLRs.

Immunoblot analysis of mature milk MFGM proteins confirmed anti-TLR1 reactivity in both term and preterm samples at 150 kDa, but if compared with colostrum, the band at 100 kDa disappeared while a new band more evident in preterm samples came out at 30 kDa. Two bands detectable in all samples appeared at 100 and 80 kDa after incubation with an anti-TLR2 antibody, while anti-TLR4 reactivity was observed at 75 kDa (see also Supplementary [Supplementary-material supplementary-material-1] and [Supplementary-material supplementary-material-1]). All samples tested showed two prevalent bands at 80 kDa and at 50 kDa, after anti-TLR6 antibody incubation.

Semiquantitative analysis of immunoreactive bands in MFGMs (data not shown), obtained after actin standardization, confirmed the differences observed between term and preterm mainly in colostral samples. Immunoblotting results show changes in the amount of actin; anyway, we used actin normalization assuming that the presence of actin in milk originates from cell residues, since actin is not a typical milk secreted protein and could represent proportionally the amount of cells from which TLRs are derived.

Colostrum of preterm donors shows higher immunoreactivity to anti-TLR1 Ab than that of term women, with two intense bands at 150 and 100 kDa. The band at 100 kDa disappeared in mature milk, while a new band at 30 kDa appeared, with higher intensity in preterm samples. Concerning colostrum, we could observe anti-TLR2 reactivity only in preterm samples, while two reactive bands at 100 and 80 kDa were detectable in each sample of mature milk tested. Interestingly, we could not observe any anti-TLR4 reactivity in colostral samples, while a band appeared in each sample of mature milk. Preterm colostral samples showed higher reactivity, at 75 and 50 kDa, to anti-TLR6 Ab too. The intensity values of these bands declined in mature milk.

### 3.3. Analysis of TLR Expression in Skimmed Milk

**Figure 6:**
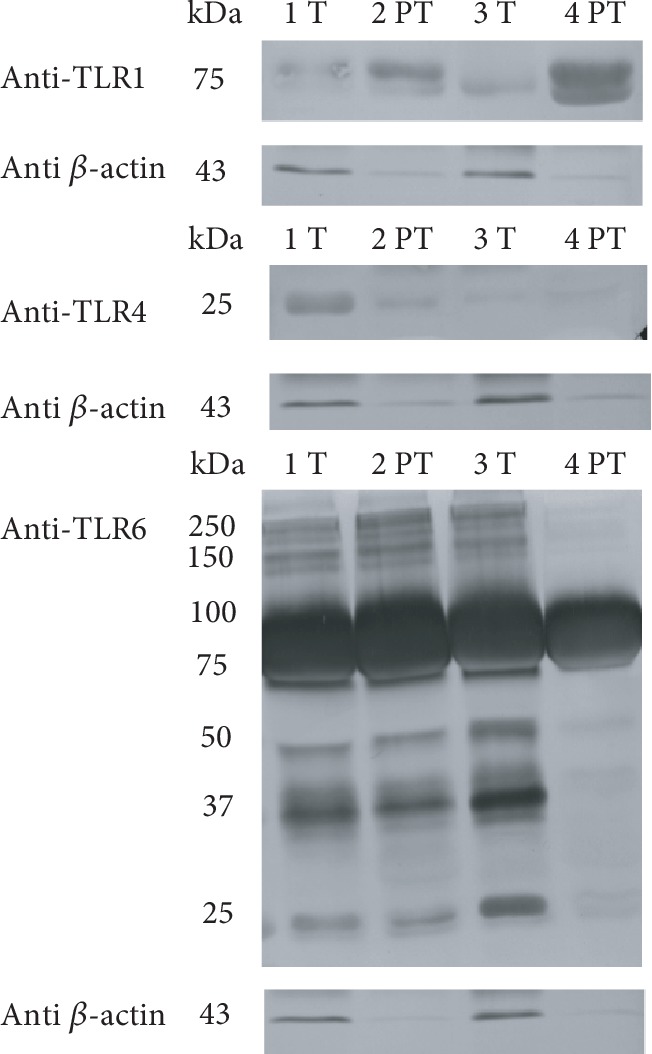
Immunoblot of TLR1/4/6 in colostral skimmed milk. Gestational age is indicated as “T” (term) or “PT” (preterm). Each number refers to a different milk sample. Standard molecular weights are indicated on the left. Each box shows the blot reactivity to a specific anti-TLR antibody and, on the lower side, the same blot incubated with anti-*β*-actin Ab.

**Figure 7:**
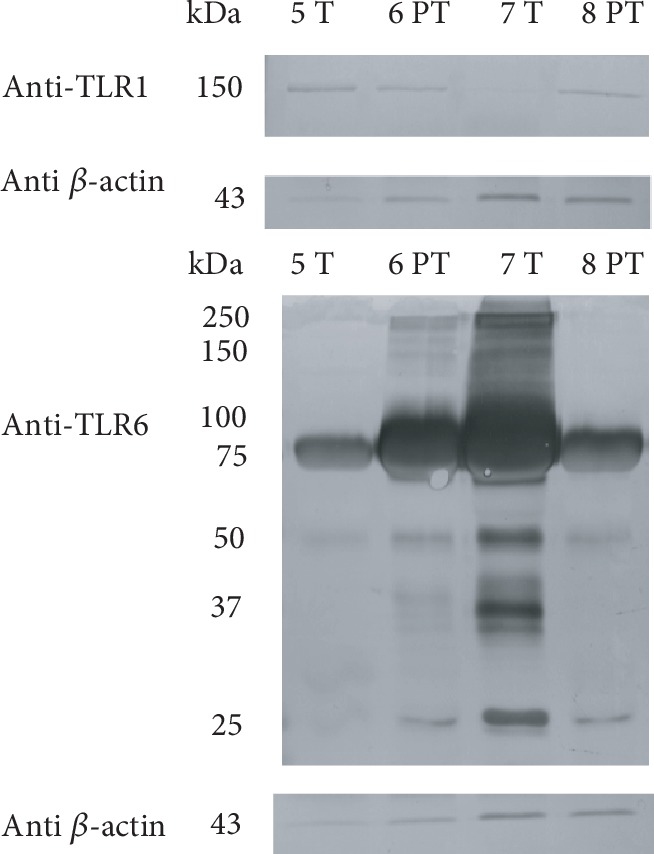
Immunoblot of TLR1/6 in mature skimmed milk. Gestational age is indicated as “T” (term) or “PT” (preterm). Each number refers to a different milk sample. Standard molecular weights are indicated on the left. Each box shows the blot reactivity to a specific anti-TLR antibody and, on the lower side, the same blot incubated with anti-*β*-actin Ab.

Immunoblots of skimmed fractions from colostrum and mature milk samples using the anti-TLR1/TLR2/TLR4/TLR6 antibodies are shown in Figures 6 and 7, respectively. Anti-*β*-actin reactivity of the same samples was used as an internal standard.

Concerning colostrum skimmed fraction, two bands around 75 kDa appeared after anti-TLR1 Ab incubation, but if compared with MFGM fraction, no bands at 150-100 kDa were observed. Anti-TLR2 and anti-TLR4 Abs determined the appearance of two bands at 75 and 50 kDa (Supplementary [Supplementary-material supplementary-material-1] and [Supplementary-material supplementary-material-1]). However, these bands were considered aspecific since the same profile was observed in samples probed with anti-*β*-actin Ab. An additional band at 25 kDa was observed after anti-TLR4 Ab incubation. The immunoreactive profile after anti-TLR6 Ab incubation is quite similar, with the appearance of high MW bands (250 kDa, 150 kDa), a most intense band between 100 and 75 kDa (the only one clearly observed in 4PT), and minor bands at 50 kDa, 37 kDa, and 25 kDa. Concerning the skimmed fraction of mature milk, the immunoreactive profile observed after anti-TLR1/2/4/6 incubation is shown in Figure 7.

A 150 kDa band appeared in all samples except in 7 T after anti-TLR1 Ab incubation. As observed in colostrum, we did not detect signals relative to TLR2 and TLR4 but only aspecific bands at 75 and 50 kDa (Supplementary [Supplementary-material supplementary-material-1] and [Supplementary-material supplementary-material-1]). Reactivity to anti-TLR6 Ab was similar to that observed for the skimmed fraction of colostrum. The main bands were observed between 75 and 100 kDa.

Immunoreactive bands against anti-TLR1 and anti-TLR4 Abs showed higher intensity in preterm than in term samples of colostrum. This trend is no more evident in mature samples, where these bands disappeared and a clear band at 150 kDa appeared only after anti-TLR1 Ab incubation. Concerning anti-TLR6 immunoreactivity, bands of higher intensity were observed in colostrum than in mature skimmed milk. Again, colostrum preterm samples revealed higher intensity bands at 75-100 kDa. This trend is no more evident in lower MW bands of both colostrum and mature skimmed milk.

The observed differences between preterm and term samples are supported by evidences reported in literature. The concentration of total protein is higher in preterm milk [15], and their expression is differently regulated. In a study on human skimmed milk [16], 28 proteins with higher expression levels and 27 proteins with lower levels were found in preterm milk compared to term milk.

Concerning TLRs, the presence of bands with apparent molecular weight lower than that of TLR mature forms (which is estimated between 87 kDa and 93 kDa considering the amino acidic sequence only) could be due to posttranslational modifications, with the production of truncated forms. In literature, there is some evidence of TLR2 and TLR4 soluble forms (sTLRs) revealed by Western blot analysis of biological samples such as saliva, amniotic fluid, plasma, and milk [5, 17, 18]. To our knowledge, the presence of soluble forms of TLR1 and TLR6 has never been reported. Immunoblot analysis of normal unstimulated whole saliva (UWS) revealed three sTLR2 polypeptides of 54, 40, and 30 kDa and four sTLR4 polypeptides of 90, 78, 54, and 44 kDa [17]. Two polypeptides (42 and 30 kDa), corresponding to the extracellular domain of the full-length TLR2 receptor (98 kDa), were found in the human amniotic fluid, where the 42 kDa isoform is the main sTLR2 released. In this compartment, sTLR2 forms were found to be constitutively expressed, but their expression level is regulated by gestational age, with high sTLR2 expression levels observed until the 37th week of gestation and decreasing levels in women who delivered at term [18]. LeBouder and colleagues [5] were the first to report the presence of soluble forms of TLR2 in human plasma and milk, where they observed up to six polypeptide bands of 83, 70, 66, 40, 38, and 25 kDa. Another study [19] reported variations to this pattern in breast milk, in terms of number and intensity of bands, with the absence of 70 and 40 kDa bands and the appearance of a 130 kDa band, probably due to sample variability and the type of antibody tested. In fact, the polyclonal (p) Ab used was found to be specific for the 83 and 38 kDa bands, whereas the monoclonal (m) Abs tested revealed two bands of 83 and 26 kDa. Moreover, pAb detected the presence of commercially available recombinant sTLR2 produced in mouse myeloma cell lines, while anti-TLR2 mAbs were unable to detect the recombinant protein. Our results differ from the previous reports, since we observed only a 25 kDa band after anti-TLR4 Ab incubation in the skimmed fraction of colostrum. The difference could be due to a number of reasons, such as the choice of antibody, which could recognize an N-term or a C-term region of TLR. The mechanism of sTLR production is still unclear, but it was suggested that sTLR2 production does not involve new protein production and is a result of posttranslational modification, probably starting from the C-terminal side [5]. The Abs chosen in this study recognize a central region of the TLR2 and TLR4 ectodomains, but part of them could be removed during the processing of soluble forms with the consequent failure of protein binding. Another possibility could be the time-related amount of soluble forms in milk, since a decline of sTLR2 levels over time postpartum was reported, with the disappearance of some soluble forms few days after delivery [5, 19]. Finally, it is possible that some signals reflect nonspecific binding of the anti-TLR2 and anti-TLR4 polyclonal Abs to proteins constitutively present in the human milk, as previously stated in [18]. In the skimmed fraction of milk, we observed two anti-TLR2/TLR4 immunoreactive bands at 75 and 50 kDa that were considered aspecific since the same reactive profile was observed after anti-*β*-actin Ab incubation.

### 3.4. Mass Spectrometry Identifications

**Figure 8:**
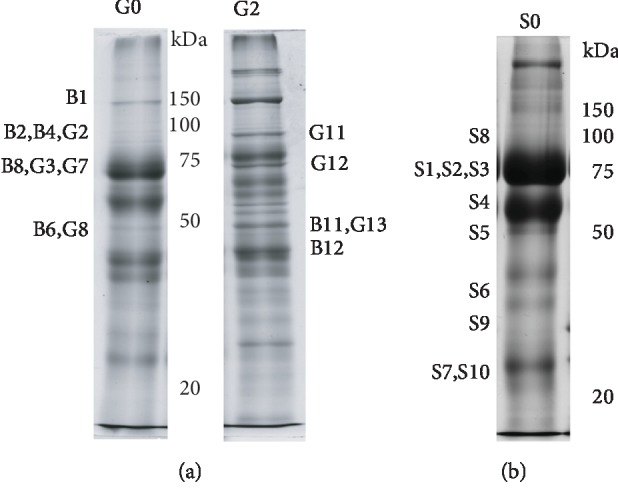
Representative image of proteins from MFGM (a) and skimmed milk (b) fractions of colostrum (0) and/or mature milk (2) after SDS-PAGE separation. The name of bands analyzed by mass spectrometry is reported beside each lane. Bands labelled with “B” were digested from PVDF blots, while “G” bands were digested from polyacrylamide gels. S1 was digested from polyacrylamide gels, and S2, S3, S4, S5, S6, S7, S8, S9, and S10 were digested from PVDF blots.

Mass spectrometry analysis of immunoreactive bands detected in MFGM and skimmed milk fractions (Figure 8) led to the identification of TLR2 and some TLR-related proteins, but the presence of TLR1/4/6 was not determined (Supplementary [Supplementary-material supplementary-material-1]). In detail, TLR2 was identified in B2, B4, G2, and G11; antigen CD14 was identified in B6, B11, B12, G12, and G13; CD36 glycoprotein was identified in G3, G8, G12, S1, S2, and B8; leucine-rich protein was identified in S3, S4, S5, G7, and G12; tenascin was identified in S1, S3, S4, S5, S6, S8, S10, B1, B2, B6, B11, B12, and G12; and zinc alpha glycoprotein was identified in S3, S4, and S5.

**Figure 9:**
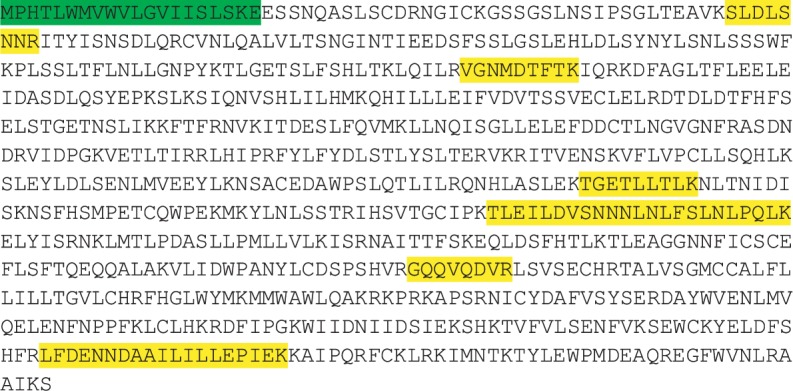
Protein sequence of TLR2. The signal peptide is highlighted in green, while identified peptides after mass spectrometry are highlighted in yellow.

TLR2 was detected by mass spectrometry in the MFGM fraction only, with peptides of six different sequences. In order to assess if TLR2 found in the MFGM fraction corresponds to the whole receptor or parts of it, the sequences of the identified peptides were overlapped to the complete sequence of TLR2. As shown in Figure 9, MS-identified peptides almost cover the entire sequence of TLR2, with four peptides belonging to the extracellular domain (SLDLSNNR: aa 56-63 corresponding to leucine-rich repeat (LRR) 1; VGNMDTFTK: aa 156-164, LRR 5; TGETLLTLK: aa 404-413, LRR 14-15; and TLEILDVSNNNLNLFSLNLPQLK: aa 458-480, LRR 17-18), one belonging to the transmembrane region (GQQVQDVR: aa 572-579, LRRCT), and one belonging to the C-terminal intracellular domain (LFDENNDAAILILLEPIEK: aa 724-742, TIR), confirming the association of the detected form of TLR2 with the MFG membrane.

Our MS results are supported by studies reporting the finding of TLR2 in MFGMs but not in serum of milk [2, 20]. Concerning other TLRs, the presence of TLR1/2/3/4/5/6/7/9 was detected at an mRNA level in cells isolated from human breast milk [21]; however, proteomic studies performed so far do not report the presence of TLR1 and TLR6 proteins in MFGM and skimmed fractions of breast milk.

It is interesting to note that our MS analysis of immunoreactive bands detected some TLR-related proteins such as CD14 and CD36 or LRR-containing proteins. Cluster of differentiation 14 (CD14) is a pattern recognition receptor with a bent solenoid structure typical of leucine-rich repeat proteins [22]. It is present in soluble form in the blood or as a GPI-anchored membrane protein on myeloid cells and interacts with TLR2 and TLR4 in ligand recognition [3]. CD14 acts as a coreceptor for TLR2/TLR6 and for TLR2/TLR1 heterodimers in response to diacylated and triacylated lipopeptides, respectively [23], and belongs to the lipopolysaccharide (LPS) receptor, a multiprotein complex containing at least CD14, MD2, and TLR4 [24]. CD36 (cluster of differentiation 36) is a multifunctional glycoprotein involved in TLR pathways. It acts as a receptor for a broad range of ligands (proteins and lipids), interacting with multiple receptors and participating in signal transduction. CD36 is involved in TLR2 activation by microbial anionic ligands such as lipoteichoic acid and TLR4/TLR6 by endogenous ligands. A model proposed in [25] suggests an interaction between CD36 and CD14, where CD36 binds to anionic ligands and transfers them to CD14 which loads them into TLR2/TLR1 or TLR2/TLR6 heterodimers. In this work, we identified CD14 in the MFGM fraction only, while other studies report its presence in the skimmed fraction too [16]. On the contrary, CD36 was detected in both MFGM and skimmed fractions of milk.

Identified proteins in skimmed milk include zinc *α*2-glycoprotein and leucine-rich *α*-2-glycoprotein. Zinc *α*2-glycoprotein (ZAG) is a 40 kDa protein secreted in body fluids, which stimulates lipid degradation in adipocytes. The exact function of ZAG in physiological conditions is still unknown. ZAG shows high sequence homology with a lipid-mobilizing factor; thus, it is considered an adipokine. However, its structure is similar to that of MHC class I antigen-presenting molecule indicating that ZAG may have a role in the immune response [26]. Leucine-rich *α*2 glycoprotein (LRG1) is a 40 kDa serum protein and the founding member of the leucine-rich repeat (LRR) family of proteins. It is involved in neutrophil degranulation, positive regulation of angiogenesis, and proliferation of endothelial cells [27, 28]. LRG1 can bind to cytochrome c, a molecule that in the extracellular space is implicated in the initiation of arthritis via the NF-*κ*B pathway, suggesting its role as a proinflammatory molecule [29]. LRG1 was found to mitigate apoptosis in lymphocytes treated with extracellular cytochrome c [30].

Leucine-rich repeats and immunoglobulin-like domains protein 1 (LRIG1) is another LRR-containing protein found in both MFGM and skimmed fractions of milk. LRIG1 is a membrane protein with a series of LRRs, three immunoglobulin-like (Ig-like) domains, one transmembrane domain, and a cytosolic tail. It is a negative regulator of signaling by receptor tyrosine kinases. LRIG1 interacts with EGFR/ERBB1, ERBB2, ERBB3, and ERBB4 family receptors through the LRR and Ig-like extracellular domains [31]. However, its physiological role is still controversial, since LRIG1 may have low affinity for EGFR and their interaction could take place only with high quantity levels of both proteins [32].

Finally, tenascin, a glycoprotein of the extracellular matrix implicated in neuronal and axonal migration during development, was identified in the MFGM and skimmed fractions. It contains EGF-like and fibronectin type III domains and can exist in homoexameric or homotrimeric forms. Tenascin is involved in various physiological processes such as cell adhesion, cellular response to vitamin D and retinoic acid, extracellular matrix organization, and positive regulation of cell proliferation.

Its expression is induced by TGF-*β*1, and it is released after cell damage of infections. In fact, tenascin quickly reacts during acute inflammatory response to LPS, through the activation of TLR4 or integrins, promoting the innate immunity response with the synthesis of proinflammatory cytokines [33]. Although its role in the mammary gland and human breast milk is still unknown, tenascin may be involved in tissue reshaping associated with pregnancy. A different glycosylation pattern of tenascin has been reported in the milk of preterm with respect to term women. Since tenascin functions depend on adhesion ability with other MEC components and membrane receptors, a different glycosylation profile may change such properties [20]. We could identify tenascin in the skimmed milk, where probably it is more represented, as demonstrated by the higher number of peptides identified, but also in the MFGM fraction. Such observation is compatible with its role as an extracellular matrix protein, which could be secreted or associated with the membrane. The presence of tenascin in the MFGM of milk has previously been reported, and it shows higher expression levels in colostrum than in mature milk [34], whereas lower levels of tenascin were detected in the skimmed milk of preterm with respect to term women [16].

### 3.5. MS Analysis of Proteins after the Modified ELISA Test

Since TLR2 was detected only in MFGM, a further test on skimmed milk fractions was performed with the modified ELISA, in order to selectively detect proteins interacting with the anti-TLRs used in this study. We limited the assay to colostrum of preterm women, since after Western blotting analyses, we observed more intense immunoreactive profiles in these samples. After anti-TLR1 Ab incubation, we detected lactotransferrin and phosphatidylinositol 4,5-bisphosphate 5-phosphatase A, a protein with two isoforms of 100 and 70 kDa (in the WB, we observed a 75 kDa band). Anti-TLR2 incubation revealed again the presence of lactotransferrin and Rho GTPase-activating protein 7 (in the WB, a 75 kDa band was observed). Anti-TLR4 interacted with zinc *α*2-glycoprotein and lactotransferrin (in the WB, 25 kDa was observed) whereas anti-TLR6 Ab incubation with skimmed milk revealed the presence of lactotransferrin and *β*-casein (WB revealed a series of bands at 100-75, 50, 37, and 25 kDa). The MS of modified ELISA eluates did not reveal any TLR. Lactotransferrin was detected in all the samples: this highly abundant protein could have “obscured” other potential targets, but this event raises the question of Ab specificity.

## 4. Conclusions

MFGM fraction from preterm colostrum showed higher reactivity to anti-TLR1, anti-TLR2, and anti-TLR6 antibodies, compared to that from term samples. In the skimmed fraction, no immunoreactivity to anti-TLR2 was observed, whereas bands at low molecular weight appeared after incubation with anti-TLR4 and anti-TLR6 antibodies. LC-MS/MS analysis of immunoreactive bands confirmed the presence of the entire membrane receptor TLR2 in the MFGM of human milk, but not in the skimmed milk. With regard to TLR1, TLR4, and TLR6, despite the observation of immunoreactive bands, their presence was not confirmed after MS analysis. However, it is interesting to note that the MS analysis of these bands allowed the identification of proteins related to TLRs, because of the presence of LRR domains in their sequence or their direct interaction with TLRs. MS analysis of the ELISA fractions did not reveal any TLR but confirmed proteins identified in immunoreactive bands. The presence of soluble forms of TLRs in human milk remains an open question. It is possible that the soluble forms of TLRs in milk reported in literature, which were observed only by immunoblot assays, are related to crossreactivity events with these proteins. Anyway, the lack of MS identification for TLR1, TLR4, and TLR6 cannot assure their total absence in milk, where their concentration may be inferior to the limit of detection of the instrument and the DDA method used in this study could have excluded them. It is clear that TLR2 is surely present in milk, and we suggest that soluble forms may be ascribed to other milk proteins sharing conformational domains capable of reacting with and modulating TLR signaling.

## Figures and Tables

**Table 1:** List of antibodies used in this study.

Antibody	Source	Producer	Dilution
Anti-TLR1	Polyclonal rabbit antibody, directed against a recombinant peptide from human TLR1 (STJ25862)	St John's Laboratory	1 : 500
Anti-TLR2	Polyclonal rabbit antibody (H-175), directed against aa 180-354 of human TLR2 (sc-10739)	Santa Cruz Biotechnology, Inc.	1 : 500
Anti-TLR4	Polyclonal rabbit antibody (H-80), directed against aa 242-321 of human TLR4 (sc-10741)	Santa Cruz Biotechnology, Inc.	1 : 500
Anti-TLR6	Polyclonal rabbit antibody directed against a peptide of 13 aa near the central part of human TLR6 (PRS3653)	Sigma-Aldrich	1 : 1000
Anti-*β*-actin	Polyclonal rabbit antibody (20536-1-AP)	Proteintech	1 : 2000
Anti-rabbit-AP	Polyclonal goat anti-rabbit antibody conjugated with alkaline phosphatase (A3687)	Sigma-Aldrich	1 : 3000

## Data Availability

The data used to support the findings of this study are available from the corresponding author upon request.

## Supplementary Materials

Supplementary Materials Table S1: list of proteins identified after mass spectrometry such as TLRs or TLR-related proteins. Figure S1: complete Western blot with anti-TLR2 for MFGM colostrum. Figure S2: complete Western blot with anti-TLR2 for MFGM mature milk. Figure S3: complete Western blot with anti-TLR4 for MFGM colostrum. Figure S4: complete Western blot with anti-TLR4 for MFGM mature milk. Figure S5: complete Western blot with anti-TLR2 for skimmed colostrum. Figure S6: complete Western blot with anti-TLR2 for skimmed mature milk. Figure S7: complete Western blot with anti-TLR4 for skimmed colostrum. Figure S8: complete Western blot with anti-TLR4 for skimmed mature milk.
