# Supplementary material for: Analysis of Toll-Like Receptors in Human Milk: Detection of Membrane-Bound and Soluble Forms
Source: J Immunol Res. 2019 Dec 4;2019:4078671. doi: 10.1155/2019/4078671 (PMC6914901; doi:10.1155/2019/4078671)

SUPPLEMENTARY MATERIAL

Table S1: list of proteins identified after mass spectrometry as TLRs or TLR-related proteins.

Table S1: list of proteins identified after mass spectrometry as TLRs or TLR-related proteins

Protein name	Band name	Sample	Donor type	Theoretical MW	Observed MW	Uniprot ID	identified peptides	protein score
TLR2	B4	Globules colostrum	healthy, pre-term	87515	100000	O60603	LFDENNDAAILILLEPIEK<br>TLEILDVSNNNLNLFSLNLPQLK	127
TLR2	G2	Globules colostrum	pre-term	87515	100000	O60603	SLDLSNNR<br>TGETLLTLK	71
TLR2	B2	Globules colostrum	term	87515	100000	O60603	GQQVQDVR	47
TLR2	G11	Globules mature	term	87515	100000	O60603	SLDLSNNR<br>VGNMDTFTK + oxidation	87
Monocyte differentiation antigen CD14	B6	Globules colostrum	term	40678	50000	P08571	ATVNPSAPR	37
Monocyte differentiation antigen CD14	B11	Globules mature	term	40678	50000	P08571	ATVNPSAPR<br>AFPALTSLDLSDNPGLGER	41
Monocyte differentiation antigen CD14	B12	Globules mature	term	40678	45000	P08571	ATVNPSAPR	64
Monocyte differentiation antigen CD14	G12	Globules mature	term	40678	75000	P08571	ATVNPSAPR	32
Monocyte differentiation antigen CD14	G13	Globules mature	term	40678	50000	P08571	QYADTVK<br>ATVNPSAPR<br>VLDLSCNR<br>AFPALTSLDLSDNPGLGER	179
CD36 Platelet glycoprotein 4	G3	Globules colostrum	term	53589	75000	P16671	TGTEVYR<br>SSMFQVR + oxidation<br>VAIIDTYK<br>QVVLEEGTIAFK<br>SQVLQFFSSDICR<br>TYLDIEPITGFTLQFAK<br>AFASPVENPDNYCFCTEK	351
CD36 Platelet glycoprotein 4	G12	Globules mature	term	53589	75000	P16671	TGTEVYR<br>SSMFQVR + oxidation<br>VAIIDTYK<br>QVVLEEGTIAF<br>SIYAVFESDVNLK<br>SQVLQFFSSDICR<br>TYLDIEPITGFTLQFAK<br>AFASPVENPDNYCFCTEK	503

**CD36 Isoform 2 of Platelet glycoprotein 4**	S1	Skimmed colostrum	term	32586	75000	P16671-2	TGTEVYR	51
**CD36 Isoform 4 of Platelet glycoprotein 4**	B8	Globules colostrum	pre-term	46631	75000	P16671-4	SIYAVFESDVNLK<br>SIYAVFESDVNLK<br>SQVLQFFSSDICR<br>TYLDIEPITGFTLQFAK	278
**CD36 Isoform 4 of Platelet glycoprotein 4**	G8	Globules colostrum	term	46631	50000	P16671-4	VAIIDTYK<br>SIYAVFESDVNLK<br>SQVLQFFSSDICR	118
**CD36 Isoform 4 of Platelet glycoprotein 4**	S2	Skimmed colostrum	pre-term	46631	75000	P16671-4	QVVLEEGTIAFK<br>SIYAVFESDVNLK<br>SQVLQFFSSDICR<br>TYLDIEPITGFTLQFAK	183
**Leucine-rich alpha-2-glycoprotein**	S5	Skimmed colostrum	term	38382	50000	P02750	CAGPEAVK<br>VAAGAFQGLR<br>ALGHLDLSGNR<br>DLLLPQPDLR<br>ENQLEVLEVSWLHGLK<br>TLDLGENQLETLPPDLLR	339
**Isoform 2 of Leucine-rich repeats and immunoglobulin-like domains protein 1**	G7	Globules colostrum	term	117958	75000	Q96JA1-2	LCAGSAYHK	31
**Isoform 2 of Leucine-rich repeats and immunoglobulin-like domains protein 1**	G12	Globules mature	term	117958	75000	Q96JA1-2	LCAGSAYHK	31
**Isoform 2 of Leucine-rich repeats and immunoglobulin-like domains protein 1**	S3	Skimmed colostrum	term	117958	75000	Q96JA1-2	LCAGSAYHK	41
**Isoform 2 of Leucine-rich repeats and immunoglobulin-like domains protein 1**	S4	Skimmed colostrum	term	117958	66000	Q96JA1-2	LCAGSAYHK	45
**isoform 2 of Tenascin**	S3	Skimmed colostrum	term	196895	75000	P24821-2	SIPVSAR<br>FTTDLDSPR<br>LDAPSQIEVK<br>ETFTTGLDAPR<br>VPGDQTSTIIQELEPGVEYFIR	193
**isoform 2 of Tenascin**	S4	Skimmed colostrum	term	196895	66000	P24821-2	CVCEQGFK<br>CPSDCHGQGR<br>VPGDQTSTIIQELEPGVEYFIR	92

**isoform 2 of Tenascin**	S5	Skimmed colostrum	term	196895	50000	P24821-2	AAIDSYR<br>SFSTFDK<br>AYAAGFGDR<br>IQALNGPLR<br>FTTDLDSPR<br>APTAQVESFR<br>ITAQGQYELR<br>EEFWLGLDNLNK<br>DTDSAITNCALSYK<br>SNMIQTIFTTIGLLYPFPK<br>DLTATEVQSETALLTWRPPR<br>TVSGNTVEYALTDLEPATEYTLR	539
**isoform 2 of Tenascin**	S6	Skimmed colostrum	term	196895	37000	P24821-2	DLTATEVQSETALLTWRPPR	34
**isoform 2 of Tenascin**	S8	Skimmed colostrum	term	196895	100000	P24821-2	SIPVSAR<br>AAIDSYR<br>AYAAGFGDR<br>CVCEQGFK<br>LDAPSQIEVK<br>ETFTTGLDAPR<br>YAPISGGDHAEVDVPK<br>GLEPGQEYNVLLTAEK<br>VPGDQTSTIIQELEPGVEYFIR<br>ETSVEVEWDPLDIAFETWEIIFR<br>DVTDTTALITWFKPLAEIDGIELTYGIK	493
**isoform 2 of Tenascin**	S10	Skimmed colostrum	term	196895	25000	P24821-2	ITAQGQYELR<br>LEELENLVSSLR	86
**isoform 2 of Tenascin**	B1	Globules colostrum	term	196895	150000	P24821-2	LSWTADEGVFDNFVLK	60
**isoform 2 of Tenascin**	B2	Globules colostrum	term	196895	100000	P24821-2	CVCEQGFK	22
**isoform 2 of Tenascin**	B6	Globules colostrum	term	196895	50000	P24821-2	FTTDLDSPR	45
**isoform 2 of Tenascin**	B11	Globules mature	term	196895	50000	P24821-2	TAHISGLPPSTDFIVYLSGLAPSIR	60
**isoform 2 of Tenascin**	G12	Globules mature	term	196895	75000	P24821-2	TVSGNTVEYALTDLEPATEYTLR<br>TAHISGLPPSTDFIVYLSGLAPSIR	121
**isoform 4 of Tenascin**	B12	Globuli maturo	term	236365	45000	P24821-4	AGTPYTVTLHGEVR<br>AHISGLPPSTDFIVYLSGLAPSIR	140
**isoform 4 of Tenascin**	S1	Skimmed colostrum	term	236365	75000	P24821-4	NLTVPGSLR<br>LDAPSQIEVK<br>LEELENLVSSLR	46

**Zinc-alpha-2-glycoprotein**	S3	Skimmed colostrum	term	34465	75000	P25311	AGEVQEPELR	64
**Zinc-alpha-2-glycoprotein**	S4	Skimmed colostrum	term	34465	66000	P25311	AYLEEECPATLR	61
**Zinc-alpha-2-glycoprotein**	S5	Skimmed colostrum	term	34465	50000	P25311	AGEVQEPELR<br>YSLTYIYTGLSK<br>EIPAWVPFDPAAQITK	134
**Zinc-alpha-2-glycoprotein**	S6	Skimmed colostrum	term	34465	37000	P25311	QDSQLQK<br>SQPMGLWR + oxidation<br>AGEVQEPELR<br>FGCEIENNR<br>WEAEPVYVQR<br>YSLTYIYTGLSK<br>AYLEEECPATLR<br>QKWEAEPVYVQR<br>YYYDGKDYIEFNK<br>QDPPSVVVTSHQAPGEK<br>QDPPSVVVTSHQAPGEK<br>EIPAWVPFDPAAQITK<br>QVEGMEDWKQDSQLQK<br>HVEDVPAFQALGSLNDLQFFR	699
**Zinc-alpha-2-glycoprotein**	S7	Skimmed colostrum	term	34465	25000	P25311	AGEVQEPELR	43
**Zinc-alpha-2-glycoprotein**	S8	Skimmed colostrum	term	34465	100000	P25311	AGEVQEPELR<br>YSLTYIYTGLSK	91
**Zinc-alpha-2-glycoprotein**	S9	Skimmed colostrum	term	34465	30000	P25311	AGEVQEPELR<br>QDPPSVVVTSHQAPGEK<br>HVEDVPAFQALGSLNDLQFFR	172

Figure S1: complete Western blot with anti TLR2 for MFGM colostrum

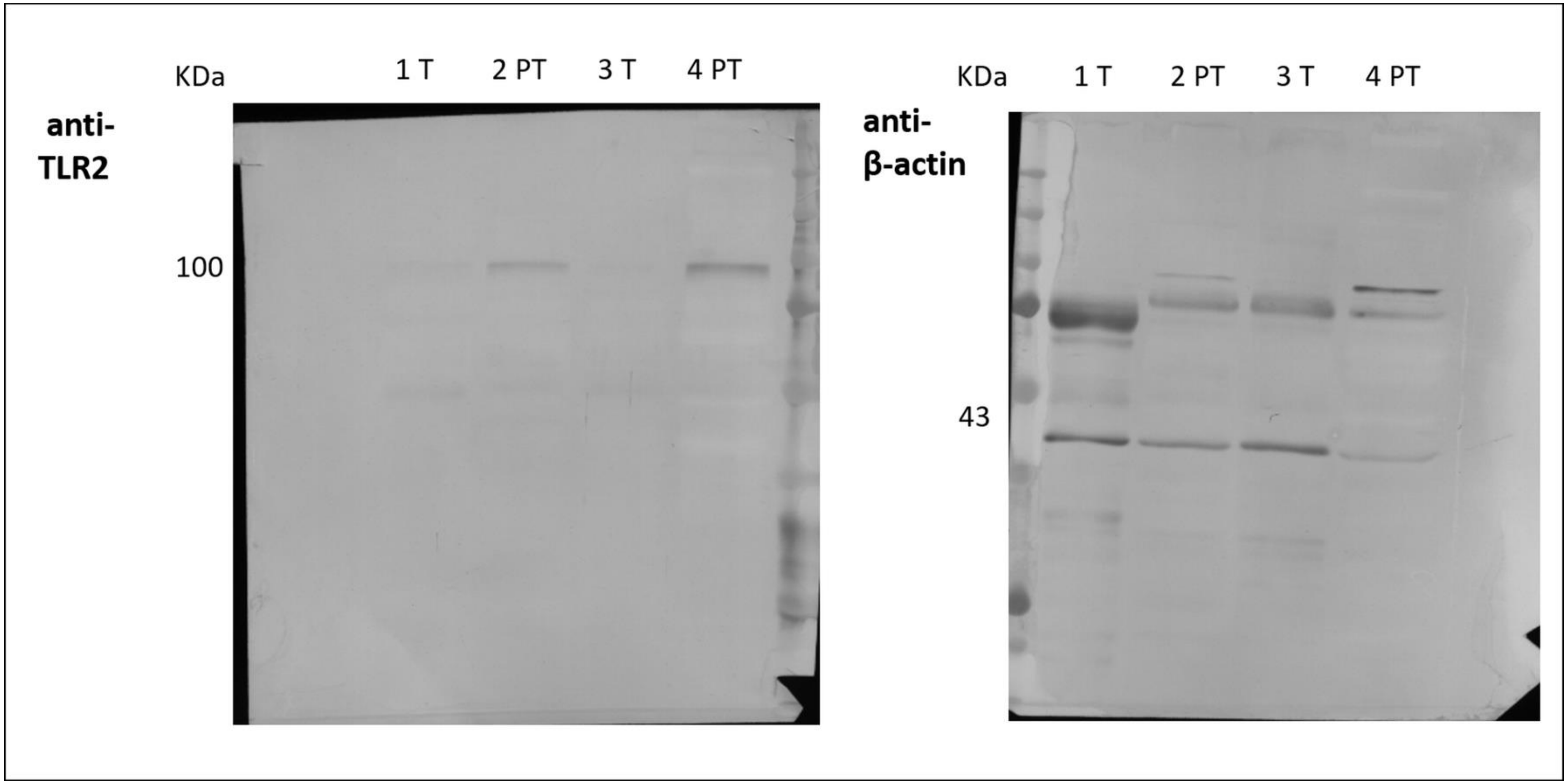

Figure S2: complete Western blot with anti TLR2 for MFGM mature milk

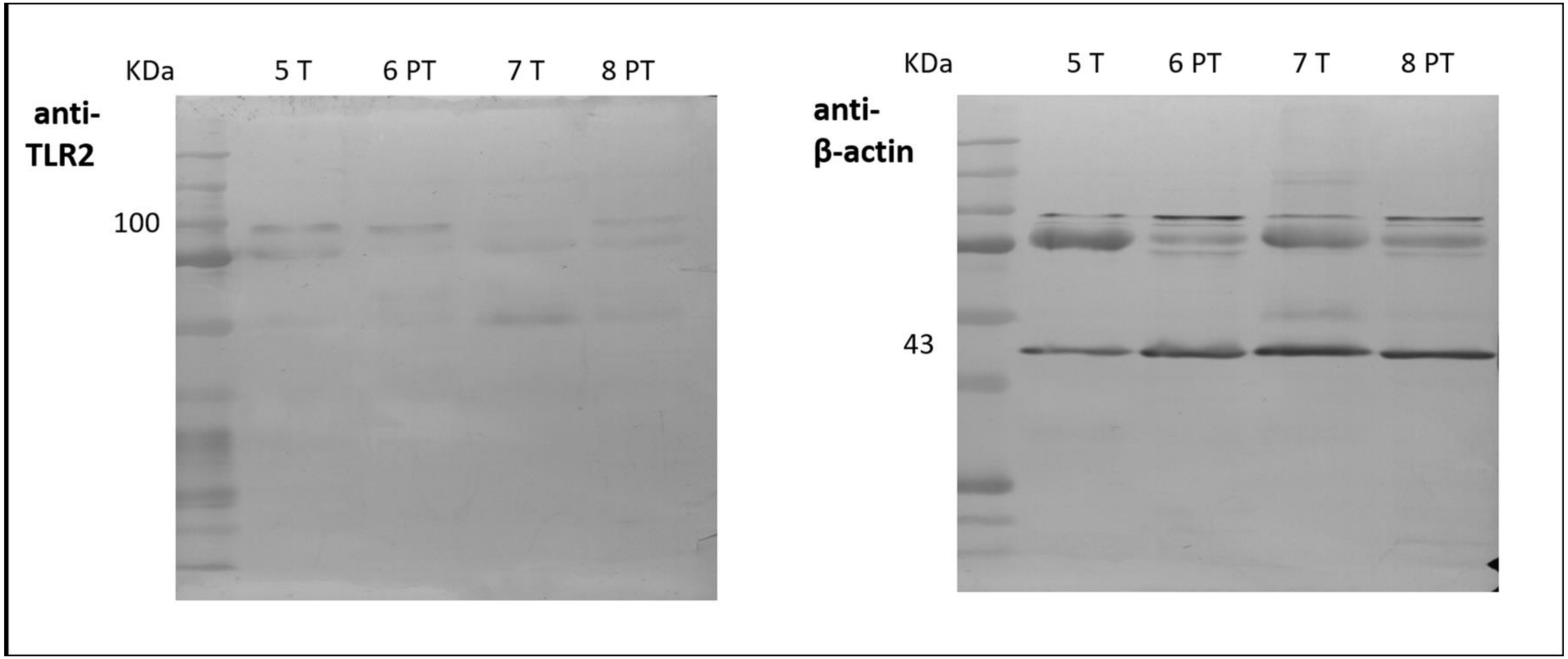

Figure S3: complete Western blot with anti TLR4 for MFGM colostrum

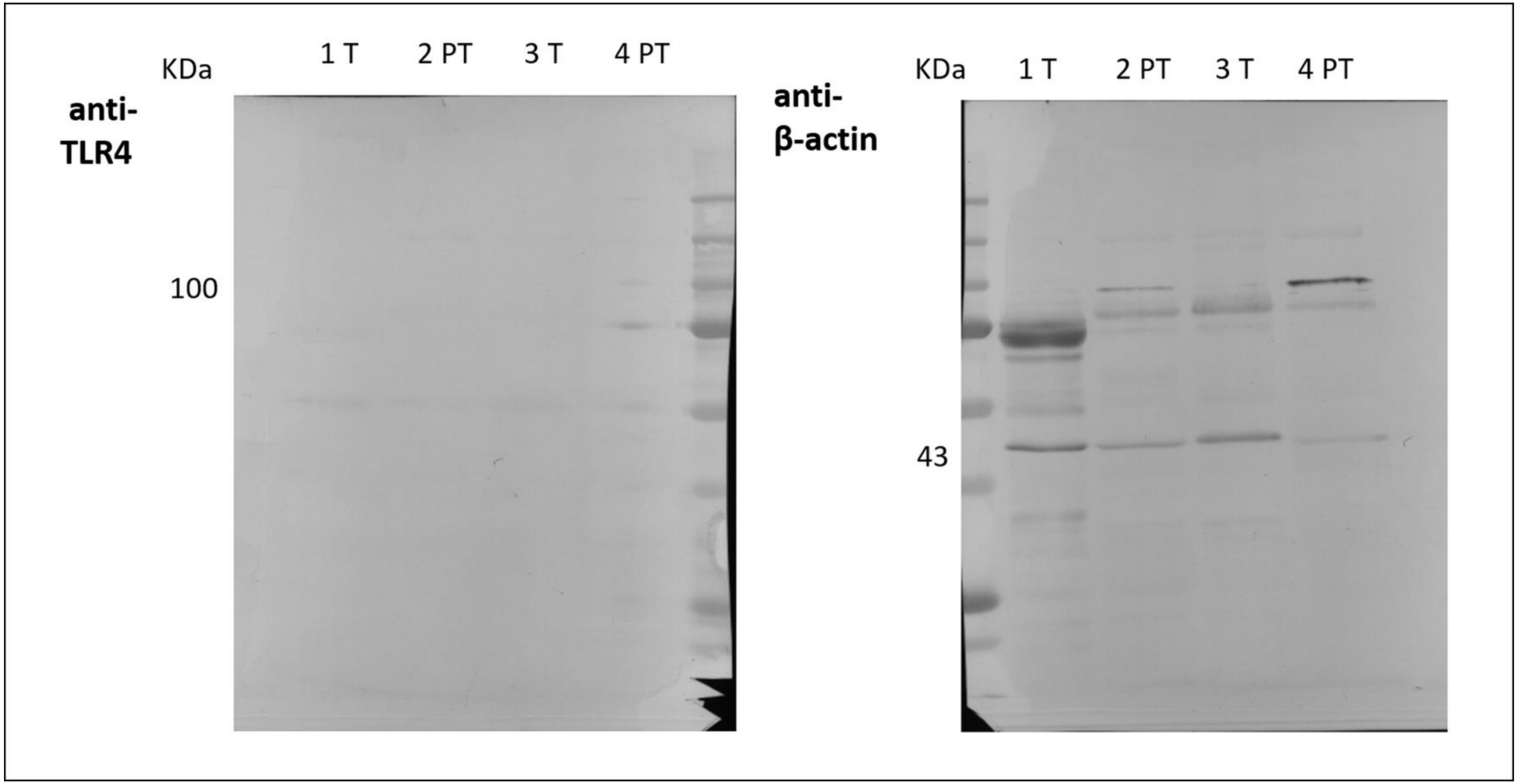

Figure S4: complete Western blot with anti TLR4 for MFGM mature milk

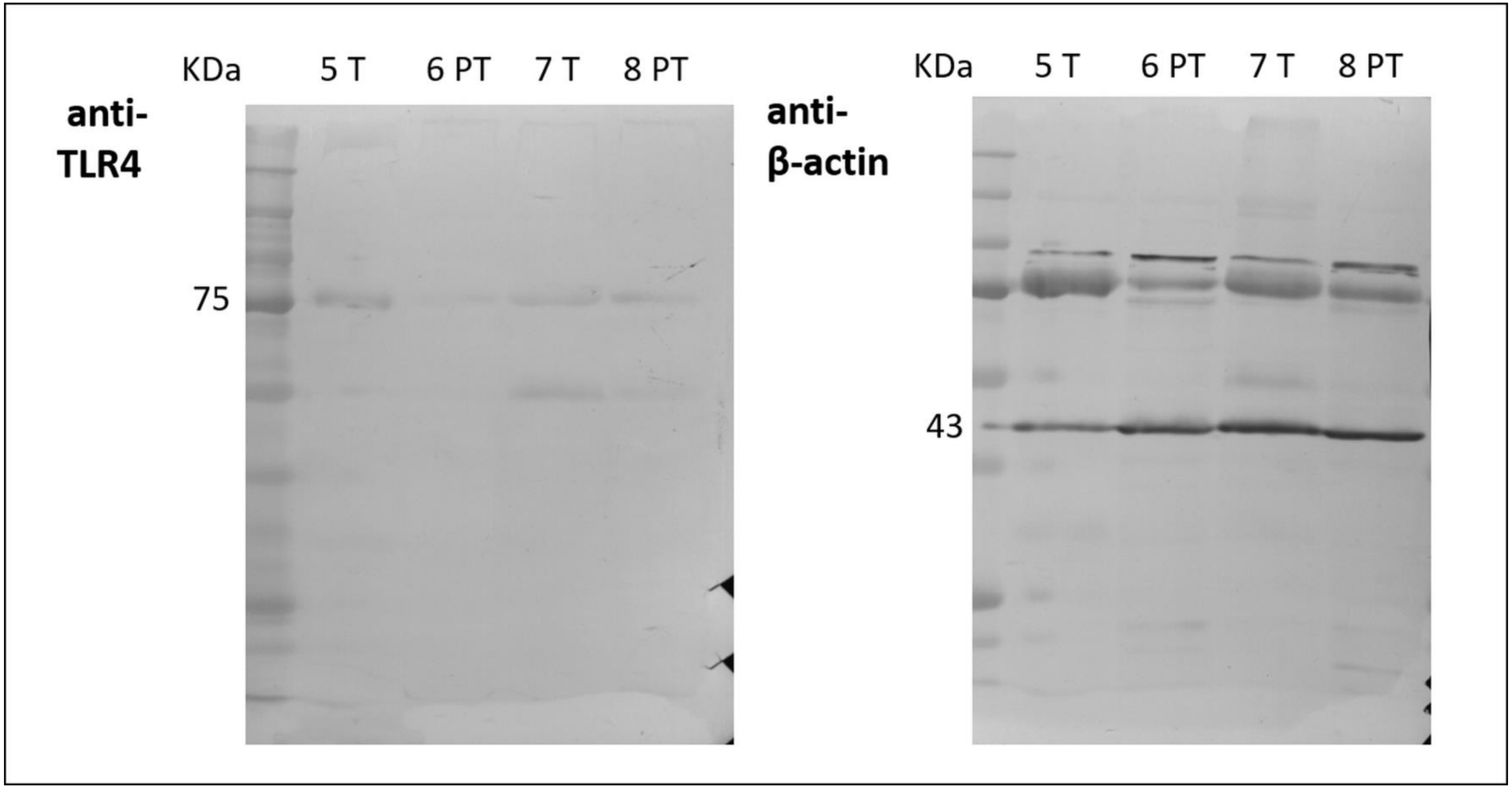

Figure S5: complete Western blot with anti TLR2 for Skimmed colostrum

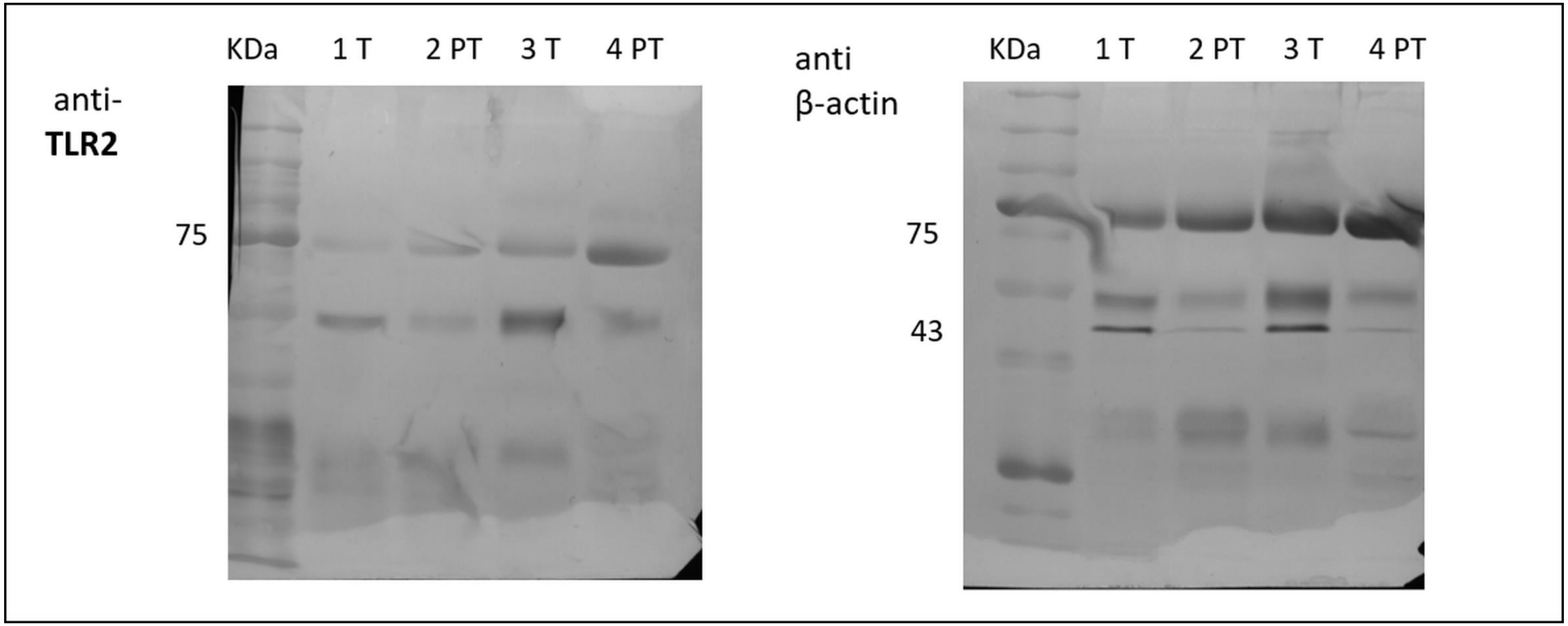

Figure S6: complete Western blot with anti TLR2 for Skimmed mature milk

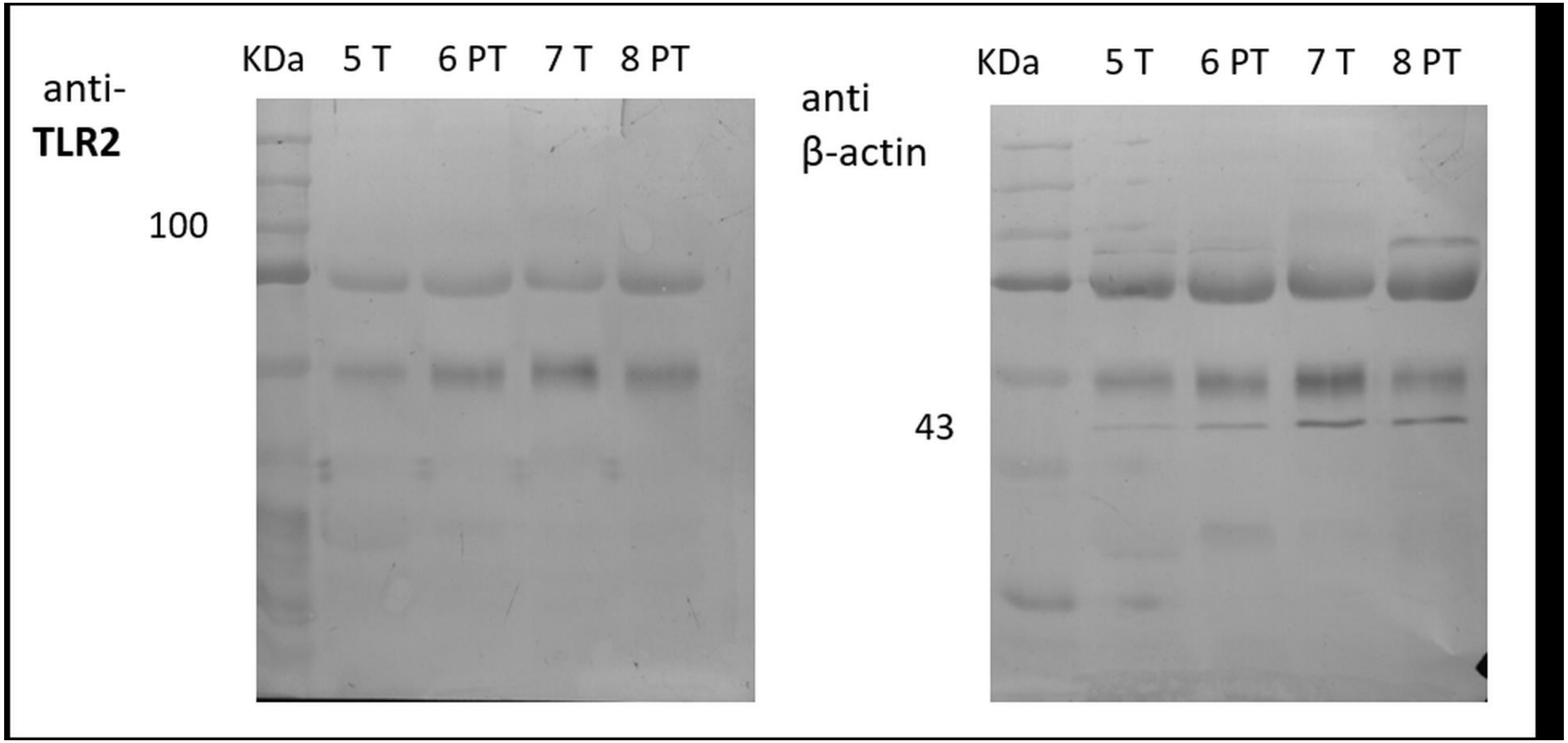

Figure S7: complete Western blot with anti TLR4 for Skimmed colostrum

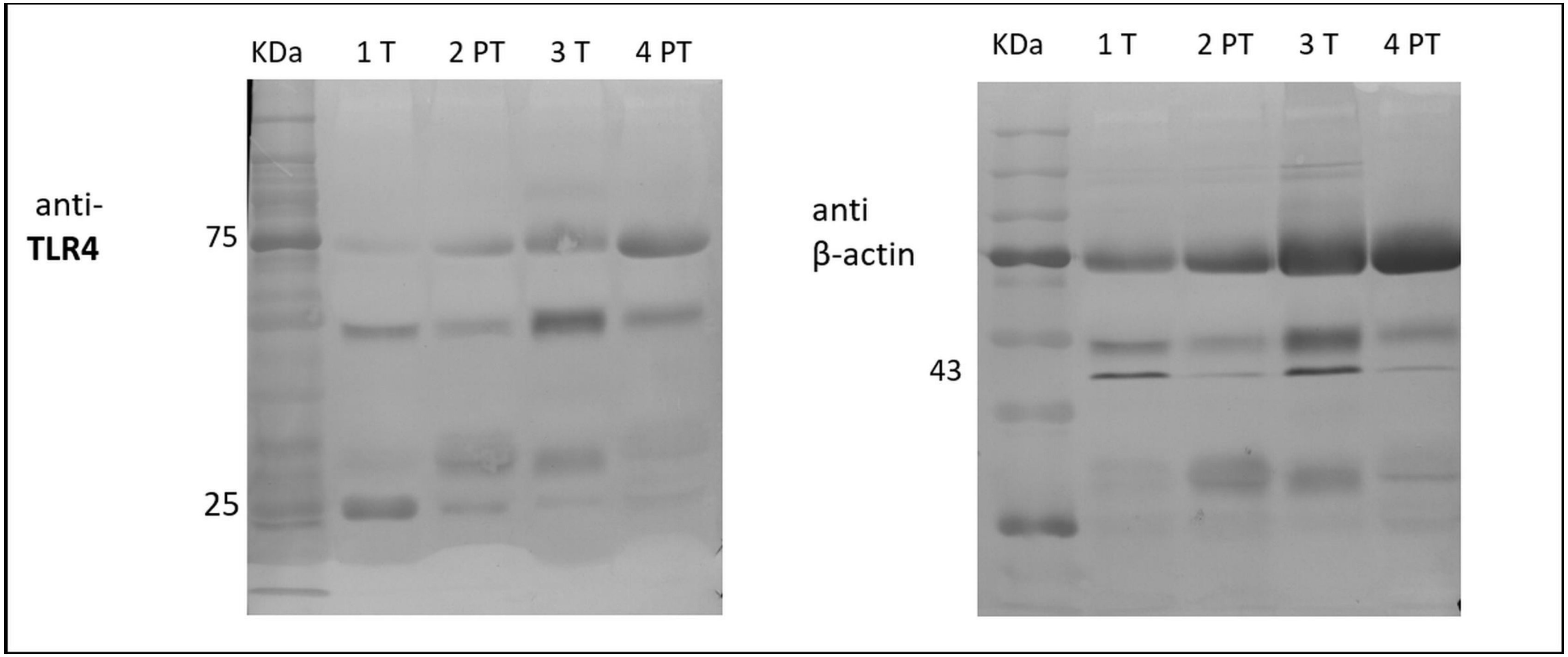

Figure S8: complete Western blot with anti TLR4 for Skimmed mature milk

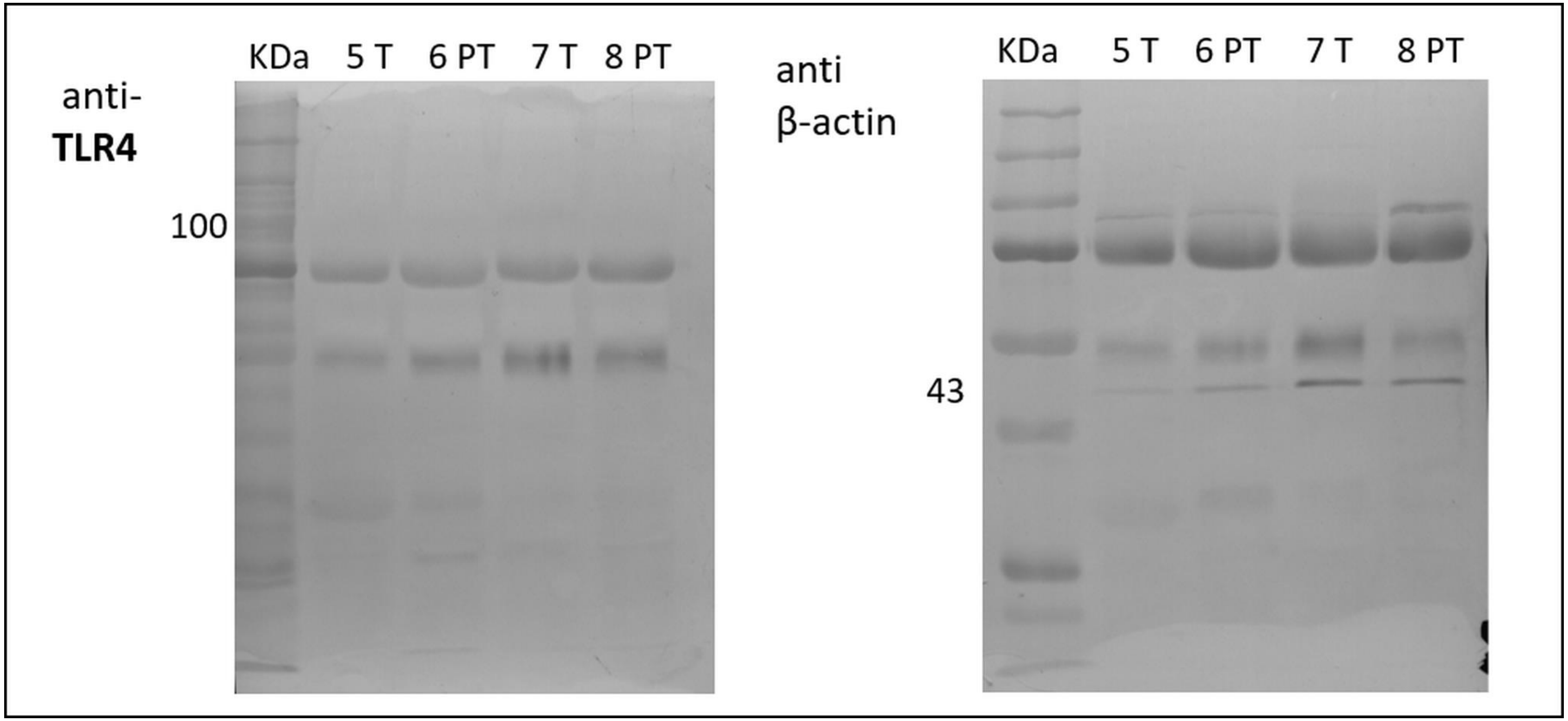

Supplement: Supplementary Materials — Table S1: list of proteins identified after mass spectrometry such as TLRs or TLR-related proteins. Figure S1: complete Western blot with anti-TLR2 for MFGM colostrum. Figure S2: complete Western blot with anti-TLR2 for MFGM mature milk. Figure S3: complete Western blot with anti-TLR4 for MFGM colostrum. Figure S4: complete Western blot with anti-TLR4 for MFGM mature milk. Figure S5: complete Western blot with anti-TLR2 for skimmed colostrum. Figure S6: complete Western blot with anti-TLR2 for skimmed mature milk. Figure S7: complete Western blot with anti-TLR4 for skimmed colostrum. Figure S8: complete Western blot with anti-TLR4 for skimmed mature milk. [file 4078671.f1.pdf]
